# Antidepressant-like Activity of Patchouli Oil var. Tapak Tuan (*Pogostemon cablin* Benth) via Elevated Dopamine Level: A Study Using Rat Model

**DOI:** 10.3390/ph15050608

**Published:** 2022-05-15

**Authors:** Puji Astuti, Khairan Khairan, Marthoenis Marthoenis, Kartini Hasballah

**Affiliations:** 1 Graduate School of Mathematics and Applied Sciences, Universitas Syiah Kuala, Banda Aceh 23111, Indonesia; puji_astuto@yahoo.co.id; 2 LLDikti Wilayah XIII, Aceh Besar 23352, Indonesia; 3 Department of Psychiatry and Mental Health Nursing, Akademi Keperawatan Tgk. Fakinah, Banda Aceh 23232, Indonesia; 4 Atsiri Research Centre, Universitas Syiah Kuala, Banda Aceh 23111, Indonesia; khairankhairan@unsyiah.ac.id; 5 Department of Pharmacy, Universitas Syiah Kuala, Banda Aceh 23111, Indonesia; 6 PT-PUI Nilam Aceh, Universitas Syiah Kuala, Banda Aceh 23111, Indonesia; 7 Pusat Riset Obat Herbal, Universitas Syiah Kuala, Banda Aceh 23111, Indonesia; 8 Department of Psychiatry and Mental Health Nursing, Universitas Syiah Kuala, Banda Aceh 23111, Indonesia; marthoenis@unsyiah.ac.id; 9 Department of Pharmacology, Faculty of Medicine, Universitas Syiah Kuala, Banda Aceh 23111, Indonesia

**Keywords:** cortisol, dopamine, patchoulol, serotonin, tail suspension

## Abstract

Essential oils are gaining popularity for their use in treating depression, including that extracted from patchouli leaves and stems (*Pogostemon cablin*). Herein, we used patchouli oil (PO) containing a high amount of patchouli alcohol derived from *P. cablin* var. Tapak Tuan. The aim of this study was to investigate the antidepressant potential of PO, with a variety of patchouli alcohol concentrations obtained from a separation process using vacuum distillation with different temperature ranges. The initial patchouli oil (iPO) was traditionally distilled by a local farmer and further distilled using a rotary evaporator at temperature ranges of 115–160 °C (POF-1); 120–160 °C (POF-2), and 125–160 °C (POF-3), resulting in products with different patchouli alcohol concentrations. POF-3, with the highest patchouli alcohol content of 60.66% (based on gas chromatography-mass spectrometry), was used for cooling crystallization, resulting in 100% patchouli alcohol crystal (pPA). A tail suspension test (TST) was performed on a rat model to screen the antidepressant potential of iPO and its derivatives. The TST results revealed that POF-3 had the best antidepressant-like effect and was second only to the fluoxetine-based antidepressant, Kalxetin^®^, where both groups had significant reductions of immobility time post-treatment (*p* < 0.0001). Other than patchouli alcohol, POF-3 also contained ledol and trans-geraniol, which have been reported for their antidepressant-related activities. Brain dopamine levels increased significantly in the group treated with POF-3 (*p* < 0.05 as compared with the control group), suggesting its primary anti-depressant mechanism. These findings suggest the potential of vacuum-distilled patchouli oil in reducing depression via dopamine elevation.

## 1. Introduction

Essential oils derived from the leaves or stems of patchouli plant (*Pogostemon cablin*), patchouli oil (PO), have become an important agro-industrial commodity [1]. Almost 90% of the world’s total PO production is from Indonesia, 70% of which originates from Aceh Province [2]. The quality of PO is commonly determined by the content of patchouli alcohol or patchoulol, where one of the leading varieties is the Tapak Tuan variety grown in Aceh Selatan Regency, Aceh Province [3]. Patchouli alcohol is often used in various commercial industries, including perfumes, cosmetics, food, and beverages [1]. The use of PO as aromatherapy is also very common, owing to its unique fragrance from patchouli alcohol and other volatile compounds [4]. The fragrance may ease stress, reduce anxiety, and boost mood [5,6]. Hence, this suggests the applicability of PO-based aromatherapy as an integrative medicine to treat depression (currently, a widely reported public health issue [7]), and particularly during the on-going coronavirus disease 2019 (COVID-19) pandemic, where depression can unanticipatedly occur in individuals and lead to feelings of worthlessness, prolonged sadness, mood disorders, and various other negative feelings [8,9].

Commercially available antidepressants have limitations related to the slow onset of action and low response rate [10]. In addition, they also have adverse side effects, such as abnormal bleeding, indigestion, and even sexual dysfunction [11]. The distressing side effects of antidepressants have inspired the exploration of other effective alternative treatments with fewer side effects [12,13]. In this regard, natural products have been investigated for their antidepressant potential and their minimal side effects [14,15]. Of which, several Indonesian essential herbs, such as lemongrass (*Cymbopogon winterianus*), *ylang-ylang* (*Canarium odoratum*), and *P. cablin*, are now being utilized as additional therapies for managing depression [4,6]. Despite the popular belief in the safety of essential oils, it is important to notice that several essential oils have been reported to cause mild-to-serious adverse effects, including impaired fetal development, bronchial hyperreactivity, and hepatotoxicity, but most of the cases were cutaneous allergic reactions [16]. In addition, a study found that *P. cablin* had low toxicity against zebrafish embryo with LC_50_ = 120 mg/L [17].

In previous studies, patchouli alcohol derived from *Valeriana wallichii* was studied for its antidepressant potential in a mice model, using a forced swim test [18,19]. The study revealed that patchouli alcohol could enhance the production of norepinephrine and dopamine [18]. A further study from the same research group revealed the involvement of the nitric oxide signaling pathway in the antidepressant mechanism of patchouli alcohol [19]. A research work from Indonesia found that encapsulation of patchouli alcohol in microcapsules could produce an antidepressant activity in ultrasonic-induced depressed mice [20]. The efficacy of patchouli alcohol as an antidepressant has also been proven in a study using mammalian target of rapamycin (mTOR), which was prior induced with chronic unpredictable mild stress (CUMS) [21]. However, we are still left with questions: “could PO have similar efficacy as an antidepressant compared with pure patchouli alcohol? Are there any other compounds in PO that could improve the efficacy of patchouli alcohol, since natural compounds tend to form synergisms with each other [22]?” To answer these questions, we need PO containing different concentrations of patchouli alcohol. By employing vacuum distillation with various temperatures ranges, we could obtain PO with various patchouli alcohol concentrations, and the follow up using cooling crystallization could produce pure patchouli alcohol [23]. To obtained the qualitative and quantitative data on compounds contained in each PO product, gas chromatography-mass spectrometry (GC-MS) was carried out [24]. Employing vacuum distillation and cooling crystallization on PO production has still been little reported. Indeed, there was a previous study investigating antidepressant activity of *P. cablin*, but they only performed a simple ethanolic extraction [25]. To test the antidepressant potential, a tail suspension test (TST) was carried out on a rat model, where plasma cortisol and brain serotonin and dopamine were also measured. Cortisol, serotonin, and dopamine are important molecules in the development of depression, becoming the targets of many antidepressants [26,27,28]. To the best of our knowledge, this is the first study to report on the efficacy of PO with different patchouli alcohol concentrations as potential antidepressants.

## 2. Results

### 2.1. Components of iPO, POFs, and pPA

**Figure 1:**
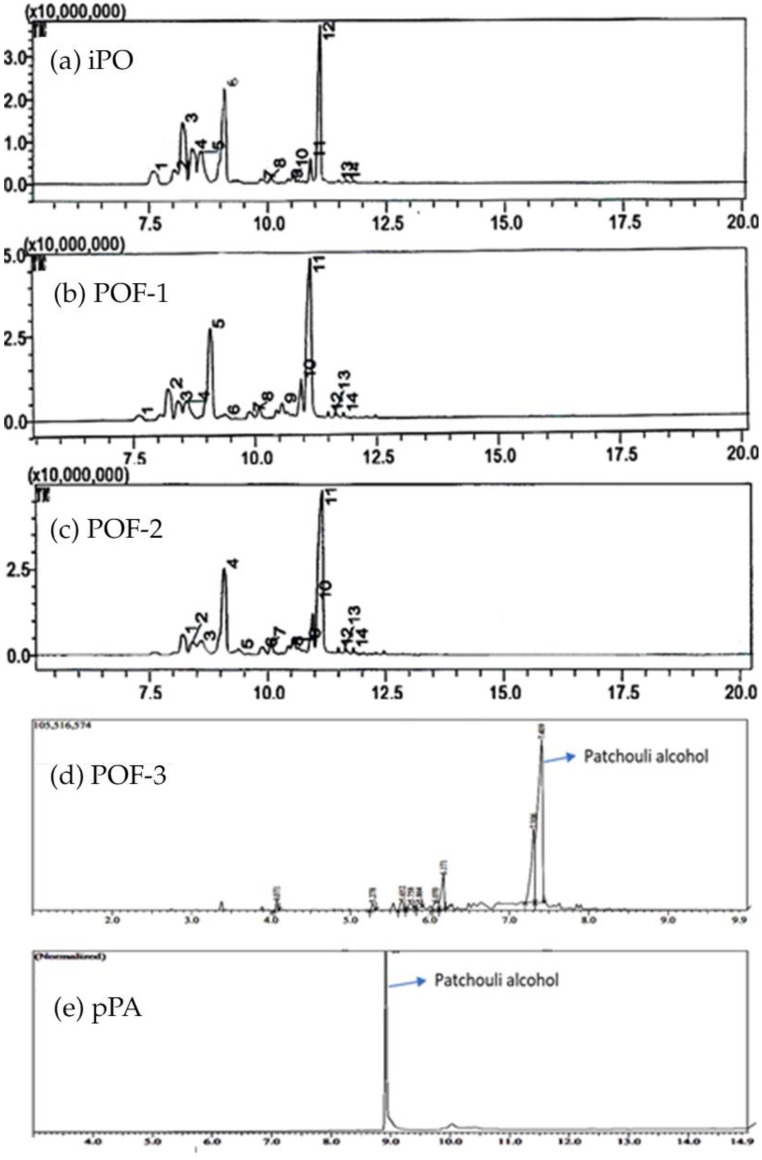
GC-MS chromatogram of iPO (**a**), POF-1 (**b**), POF-2 (**c**), POF-3 (**d**), and (pPA) (**e**).

Chemical compounds contained in the iPO, POF-1, POF-2, POF-3, and pPA were detected based on their appearance in the chromatogram, and quantitatively analyzed based on their respective spectral area; these data are presented in Table 1. Patchouli alcohol, the main component of patchouli oil, was observable with increasing concentration in samples, from iPO (28.68%), POF-1 (42.81%), POF-2 (49.29%), POF-3 (60.66%), to pPA (100%). The second largest component was δ-guaiene, contained in iPO, POF-1, and POF-2. Along with δ-guaiene; α-guaiene; 2-methyl-3-isopropenylcyclohexanol; and 2(1H)naphthalenone,3,5,6,7,8,8a-hexahydro-4,8a-dimethyl-6-(1-methylethenyl)- were compounds consistently appearing in iPO, POF-1, and POF-2, but no longer detectable in POF-3 and pPA. These compounds might have been removed by the distillation process. However, there were compounds that was only found only after the distillation, such as (-)-globulol; champacol; 8-isopropenyl-1,5-dimethyl-cyclodeca-1,5-diene; but-3-enal 2-methyl-4-(2,6,6-trimethyl-1-cyclohexenyl)-; and aromadendrene, dehydro-. Ledol was the only compound detected in the fraction resulting from 125–160 °C distillation (POF-3); and in fact, it was the second most predominant compound in the sample. Other than ledol, the compounds γ-elemene, α-gurjunene, α-selinene, (-)-β-elemene, and trans-geraniol occurred solely in a fraction resulting from 125–160 °C distillation (POF-3). The emergence of new compounds or their loss could be attributed to the change of concentration, concomitant to the distillation-based purification. Secondary transformation, which took place during the distillation process, could also be responsible for the different chemical profiles in each sample [29]. To better observe the results of the GC-MS analysis, we have presented the chromatogram of iPO, POF-1, POF-2, POF-3, and pPA in Figure 1.

### 2.2. Effect of iPO, POFs, and pPA on Depression-Related Behavior

**Figure 2:**
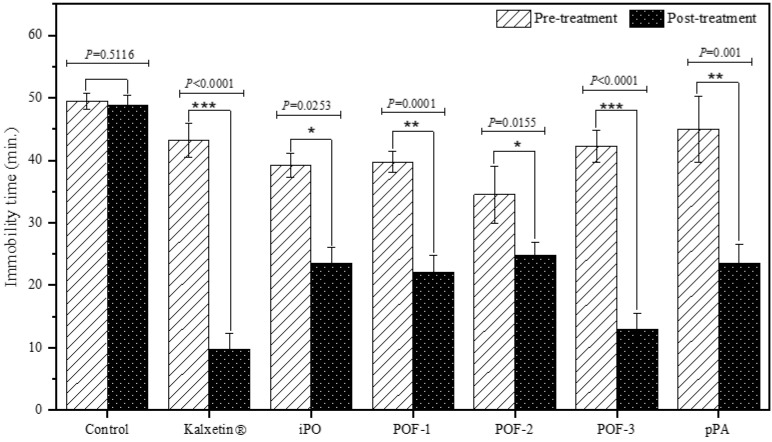
Rat’s immobility time observed during the TST, pre- and post-treatment. * significant at *p* < 0.05, ** significant at *p* < 0.01, and *** significant at *p* < 0.001 based on unpaired *t*-test with Welch’s correction.

The depressant-like activity of iPO, POFs, and pPA was determined based on their ability for reducing rat’s immobility time during TST, and the results are presented in Figure 2. The Kalxetin^®^ group experienced a dramatic reduction of immobility time of around 77.46% with a high statistical significance (*p* < 0.0001). The treatment using iPO resulted in a significant reduction (*p* = 0.0253), but it only reached 40.13%. Rats treated with POF-1 and POF-2 had immobility time reductions of around 44.65 and 28.26%, respectively. Administration of POF-3 through the inhalation route could significantly reduce the immobility time (*p* < 0.0001) of around 69.23%, which is the closest value to that obtained in the Kalxetin^®^ group. The sample with 100% patchouli alcohol purity, pPA, also yielded a statistically significant reduction (*p* = 0.001), reaching 47.78%, but it was not as high as that obtained in the POF-3 group.

### 2.3. Effect of iPO, POFs, and pPA on Plasma Cortisol

The plasma cortisol and brain serotonin and dopamine levels of all studied groups were quantitatively determined, with the results presented in Table 2. Increased cortisol level is positively correlated with depression [30]. Therefore, a reduction of its level is expected with treatment with anti-depressants. Cortisol level was observed to increase greatly in the control group, with *p*-value of 0.0073, compared with the normal group. The change of plasma cortisol was no longer statistically significant in the other groups observed. The Kalxetin^®^ group had an increased level of cortisol and almost reached a statistical significance (*p* = 0.0522) when compared with the control. The increase in cortisol level observed in the Kalxetin^®^ group might be associated with its administration via oral route with the help of feeding tube, which might have resulted in additional stress to rats [31]. Regardless, the statistical significance, POF-3 appeared to cause the highest reduction of plasma cortisol (33.17%) among the other treated groups.

### 2.4. Effect of iPO, POFs, and pPA on Neurotransmitters

Levels of neurotransmitters, serotonin and dopamine were calculated and are presented in Table 2. Increased serotonin and dopamine following the treatment should suggest an anti-depressant effect [32]. The serotonin levels observed between the normal and control groups were not statistically significant (*p* = 0.4177). Despite the Kalxetine^®^ mechanism of action, which increases the presence of serotonin [33], in this study, this group experienced a significant reduction of brain serotonin (*p* = 0.0172). This could also be possibly associated with the stressful administration of Kalxetin^®^ in the rat model [31]. Significantly lower brain serotonin level was also found in POF-2 (*p* = 0.0309) as compared with the control. It is worth mentioning that iPO, POF-3, and pPA had increased serotonin levels, but without statistical significance.

A significant reduction of brain dopamine level was observed in the control group (*p* = 0.0025). The Kalxetin^®^ group had higher dopamine level than that of the control group, but it did not reach statistical significance (*p* = 0.36). As expected, rats receiving POF-3 treatment experienced a significant elevation of dopamine level in the brain (*p* = 0.0155), in which the level was 22.56% higher than that of control group. Interestingly, a significantly higher level of brain dopamine (*p* = 0.0374) was also observed in a group treated with iPO. Further dopamine depletion, however, was obtained from the brain of rats in the POF-2 group, with statistical significance (*p* = 0.0492) compared with the control group.

## 3. Discussion

The use of essential oils via inhalation for treating depression has been widely accepted and practiced in society [34,35]. Patchouli oil’s main component, patchouli alcohol, was shown to possess antidepressant-like effects by inhibiting autophagy, repairing synapse, and restoring autophagic flux in stressed rats’ brain via mTOR signaling pathway activation [21]. Encapsulated patchouli alcohol has been reported as an effective antidepressant agent in a rat model [20]. Herein, we assessed the antidepressant-like properties of patchouli oil and its purification derivatives based on TST using a rat model. TST itself has been well-acknowledged as a valid screening method for antidepressant candidates, by observing the change of acute depressive behavior of the animal model [36].

In this present study, we found that groups treated with PO products had a significant reduction of immobility time, suggesting their antidepressant-like activities. The highest reduction was found in the group treated with POF-3, which contained 60% patchouli alcohol. The increased immobility time reduction was not dependent on the concentration of patchouli alcohol in the PO products. Even when the concentration of patchouli alcohol was 100% (pPA), the antidepressant-like effect became less defined than that of POF-3. Nonetheless, we could roughly assume that 47.78% of the total 69.23% immobility time reduction in POF-3 was contributed by patchouli alcohol, suggesting the patchouli alcohol as the dominant contributor of the antidepressant-like activity; in agreement with a previous report, where encapsulated patchouli alcohol effectively reduced the immobility time of ultrasonic-induced depressed mice (*Mus musculus*) [20]. The higher reduction in rats’ immobility times could also be attributed to the presence of other secondary metabolites, such as ledol and trans-geraniol. Ledol is among the dominant components in *Peperomia serpens* essential oil, and reported for its significant peripheral antinociceptive activity [37]. As for trans-geraniol, its antidepressant, antinociceptive, and neuroprotective activities have been reported previously [38,39,40]. There is also a possibility that these compounds could form a synergism with patchouli alcohol, as observed in other natural compounds [22,41].

Herein, POF-3 reduced the immobility time (mean ± standard deviation) from 42.25 ± 2.63 min to 13 ± 2.45 min. As a comparison, a previous research study reported the antidepressant-like effect of *Piper nigrum* Linn.-derived essential oils, where the shortest immobility time was around 40 min [42]. Another investigation on essential oils from *Lippia sidoides* achieved an immobility of 40.0 ± 10.31 min [43]. Essential oils of *Tagetes minuta* were reported in a study with the ability to reduce the immobility to no less than 50 min [44]. Taken together, the POF-3 obtained in this study could have superior antidepressant-like effects compared with essential oils derived from other plants.

In addition to immobility time, plasma cortisol and brain serotonin and dopamine were analyzed. We found that the TST performed in rats (control group) could significantly increase the cortisol level and reduce dopamine levels. Previously, a study using a non-human primate found cortisol to be strongly related to stress and depressive behavior [30]. The level of cortisol hormone significantly increases following stress or depression stimulus [45,46]. Reduction in dopamine was also possibly caused by depression [47,48]. As in the case of serotonin in this study, the level decreased in the control group, but the difference was not statistically significant. Based on previously published reports, serotonin has commonly been found to be reduced during anxiety or depression states [49,50].

Herein, there were no meaningful changes of plasma cortisol and brain serotonin observed in the treated groups. Nonetheless, significant increases of dopamine levels were obtained in the iPO and POF-3 groups, in which the latter was more significant. This finding suggests that the antidepressant activities of iPO and POF-3 were derived from elevated dopamine production. In the pPA group, the enhancement of dopamine level obtained was insignificant, suggesting that this mechanism is not correlated with patchouli alcohol. Thus, the presence of other secondary metabolites is important, to induce an antidepressant-like activity. Improved dopamine levels in the brain tissue indicate that the inhalation of essential oils could directly stimulate the rat’s brain [7]. Dopamine promotion has been assigned as the mechanism of several antidepressants [26,27]. Similarly, a previous study reported that levels of dopamine, along with norepinephrine, were elevated following a chronic treatment using *Valeriana wallichii*-derived essential oils containing predominantly patchouli alcohol (70%) and other minor volatiles (such as δ-guaiene, seychellene, viridiflorol) [18]. Even though the pure patchouli alcohol in this present study was proven unable to increase dopamine levels, the data (along with those from the previous studies) suggest that its presence as a major compound is essential. Further studies are warranted to investigate the effect of patchouli alcohol and its synergisms with other volatiles in the regulation of dopamine. It is worth noting that an antidepressant that works by inducing dopamine production was reported to have a faster onset compared to that of selective serotonin re-uptake inhibitors [26]. Cortisol, serotonin, and dopamine are intercorrelated, in which serotonin-induced production of dopamine might inhibit the production of cortisol [28]. Hence, it is understandable that both iPO and POF-3 in this present study had the lowest cortisol level among the treated groups. The foregoing association between cortisol and dopamine further suggests the involvement of dopamine in the antidepressant-like activities of iPO and POF-3.

## 4. Materials and Methods

### 4.1. Materials

This study used non-graded Aceh patchouli oil (PO) var. Tapak Tuan (*Pogostemon cablin* Benth), obtained from Atsiri Research Center, Universitas Syiah Kuala (Banda Aceh, Indonesia), which was traditionally distilled by local farmers using a set of simple steam boilers and old drums. Pharmaceutical grade Kalxetin^®^ (containing fluoxetine hydrochloride) was used and purchased from Kalbe Farma (Jakarta, Indonesia).

### 4.2. Study Design

Investigations on the antidepressant-like activity of PO and its derivatives were conducted in an in vivo study using *Rattus norvegicus*. A tail suspension test (TST) was used to study the potential attenuation of depression-related behavior in the animal model, where the subjects were subjected to an inescapable but moderately stressful situation. A total of 32 male rats (*R. norvegicus*) with body weights ranging from 180 to 250 g were employed. The animals were procured from the Faculty of Veterinary Medicine, Universitas Syiah Kuala (Banda Aceh, Indonesia). Upon retrieval, the rats were acclimated for seven days at 23–28 °C with ad libitum access to food and water and 12-h light and dark cycles. Afterward, rats were randomized and evenly distributed (n = 4) into eight groups. The first group was assigned as the normal control group, receiving neither TST nor anti-depressant treatment. The following two groups were assigned as negative and positive controls, receiving TST and TST + fluoxetine-based antidepressant (Kalxetin^®^), respectively. Kalxetin^®^ was administered via oral gavage with a dose of 20 mg per kg body weight sid. The rest of the studied groups received TST along with a variation of patchouli oil-based antidepressant candidate, namely initial patchouli oil (iPO), PO fraction-1 (POF-1), PO fraction-2 (POF-2), PO fraction-3 (POF-3), and purified patchouli alcohol (pPA). Other than immobility time (from TST), levels of cortisol, serotonin, and dopamine were also measured. This research protocol was approved by the Veterinary Ethics Committee, Faculty of Veterinary Medicine, Universitas Syiah Kuala—Banda Aceh, Indonesia (registration number: 71/KEPH/XII/2020).

### 4.3. Preparation of POFs and pPA Crystal

**Scheme 1:**
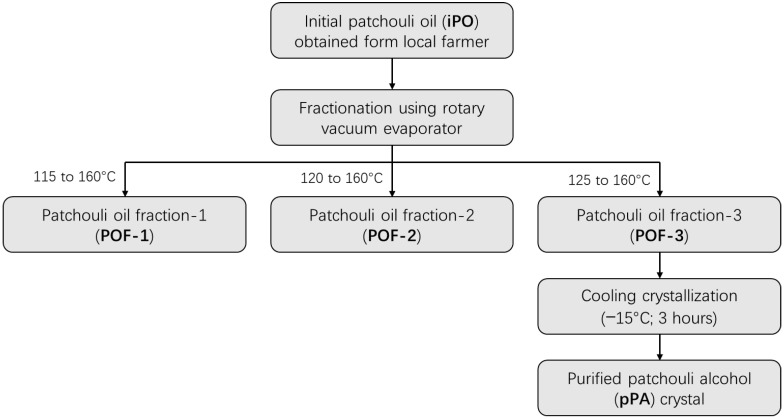
Fractionation of initial patchouli oil (iPO), and its purification into purified patchouli alcohol (pPA) crystal.

A rotary vacuum evaporator was employed and operated under 200 kPa, to separate patchouli oil into three fractions at the following temperatures: 115–160 °C (POF-1); 120–160 °C (POF-2), and 125–160 °C (POF-3). This vacuum distillation procedure was adopted from previous research [23]. All fractions were analyzed on a Shimadzu GC-MS QP 2010 Ultra (Kyoto, Japan) to determine their respective patchouli alcohol (PA) contents. The fraction with the highest PA content (POF-3) was further purified using the cooling crystallization method. About 60 g of POF-3 was poured into a beaker glass and subsequently added with diethyl ether, stirred at 50 rpm and 25 ± 1 °C (room temperature) for 2 h. Thereafter, the distillate was placed in a freezer (−15 °C) for the cooling process, which lasted a few days (±3 days) until a crystal clump appeared. GC-MS and Fourier-transform infrared (FT-IR—Shimadzu Prestige-21, Kyoto, Japan) were carried out on the produced crystal to confirm the purity of PA content. The crystal was then labeled as pPA. A schematic diagram for the preparation of POFs and pPA is presented in Scheme 1.

### 4.4. TST and Exposure of PO-Based Aromatherapies

The TST protocols used in this study followed suggestions from a previously published report [51]. The procedure was performed in a 70 × 60 × 60 cm^3^ wooden box, where ±1 cm adhesive tape was applied on the tip of the tail to hang the rat. The rat’s mobility or immobility was observed as an indicative depressed behavior every 6 min within a 60-min duration. TST was carried out twice; the first was assigned as a pre-treatment phase and the second as a post-treatment phase.

In the days after the first TST, treatment groups received inhalational administration of iPO, POFs, and pPA. Samples of iPO and POFs were first place drop-wise (5 drops) into 40 mL distilled water. In the case of pPA, 0.1 g of its crystal was dissolved using 20-min sonification in the same volume of distilled water. Each dissolved sample was inserted into an aromatherapy diffuser, which was then placed and turned on in each experimental cage for 5 min. The rat was then put into the cage which had been evenly spread with the PO-based aromatherapy. This procedure was repeated daily for 14 consecutive days prior to the second TST.

### 4.5. Determination of Plasma Cortisol and Brain Serotonin and Dopamine

Upon the completion of the second TST, all subjects were euthanized by cardiac puncture after 20-min chloroform exposure. The rat’s blood was withdrawn from the heart and centrifuged for 15 min at 3000 rpm to extract the blood plasma. Hormone cortisol level was determined using a DRG Cortisol Enzyme-Linked Immunosorbent Assay (ELISA) kit (DRG Instruments GmbH, Marburg, Germany). Each rat’s brain was immediately dissected, placed on a glass plate, and stored at −80 °C. Levels of brain serotonin and dopamine were determined using the respective ELISA kits for rat (BIOENZY, Jakarta, Indonesia).

### 4.6. Statistical Analysis

The results were expressed as the mean ± standard deviation (SD). First, the data were assessed for their normal distribution employing a Shapiro–Wilk test. Afterward, statistical significance was obtained with an unpaired *t*-test, following Welch’s correction. Herein, statistical analyses were performed on Graphpad Prism ver. 9.0.0 (San Diego, CA, USA).

## 5. Conclusions

Essential oil extracted from *P. cablin* by a local farmer in Tapak Tuan (Aceh, Indonesia), iPO, had an effective antidepressant-like activity, based on immobility time during the TST. Distillation of the essential oil using a temperature range between 125 and 160 °C, POF-3, improved the antidepressant-like activity. Patchouli alcohol was found to be the main contributor of the antidepressant-like activity of the essential oil. Both iPO and POF-3 could significantly enhance the dopamine level in the brain tissues of acutely depressed rats. However, pure patchouli alcohol could not significantly increase the dopamine level or decrease the cortisol level. Hence, other molecules in either iPO or POF-3 could be responsible for their antidepressant-like activities correlated with dopamine induction. More studies are required to fully elucidate the role of patchouli alcohol and its synergism with other molecules in regulating depression-related neurotransmitters. Considering the TST results and increased dopamine levels, POF-3 was chosen as the best potential antidepressant agent.

## Figures and Tables

**Table 1:** Results from GC-MS analysis on iPO, POFs, and pPA.

Chemical Compounds	Percentage of Area (%)				
	iPO	POF-1	POF-2	POF-3	pPA
Patchouli alcohol	28.68	42.81	49.29	60.66	100
δ-Guaiene	24.87	25.56	27.51	-	-
Ledol	-	-	-	21.54	
α-Guaiene	16.89	8.73	4.58	-	-
Seychellene	8.32	3.99	0.91	1.71	-
γ-Elemene	-	-	-	7.83	
α-Patchoulene	7.29	-	-	1.43	-
β-Patchoulene	5.05	-	-	-	-
(-)-Globulol	-	5.77	6.42	-	
Champacol	-	3.05	1.53	-	-
Globulol	2.94	-	1.85	-	-
α-Gurjunene	-	-	-	2.54	-
α-Selinene	-	-	-	2.00	-
Caryophyllene	1.99	-	0.78	-	-
8-Isopropenyl-1,5-dimethyl-cyclodeca-1,5-diene	-	1.78	-	-	-
But-3-enal, 2-methyl-4-(2,6,6-trimethyl-1-cyclohexenyl)-	-	1.75	1.96	-	-
Aromadendrene, dehydro-	-	1.48	1.23	-	-
(-)-β-Elemene	-	-	-	1.25	-
2,3,3-Trimethyl-2-(3-methyl-buta-1,3- dienyl)-cyclohexanone	1.10	-	-	-	-
Longipinocarveol, trans-	1.08	-	2.34	-	-
trans-Geraniol	-	-	-	1.05	
(-)-Globulol	0.82	1.81	-	-	-
(-)-Spathulenol	0.41	1.95	-	-	-
2-Methyl-3-isopropenylcyclohexanol	0.34	0.50	0.60	-	-
2(1H)Naphthalenone, 3,5,6,7,8,8a- hexahydro-4,8a-dimethyl-6-(1- methylethenyl)-	0.22	0.43	0.45	-	-
Selina-6-en-4-ol	-	0.39	0.55	-	-

(-) indicates the absence of the compound.

**Table 2:** Level of plasma cortisol and brain serotonin and dopamine (n = 32).

Group	Cortisol		Serotonin		Dopamine	
	Level, Mean ± SD (ng/mL)	*p*-Value	Level, Mean ± SD (ng/mL)	*p*-Value	Level, Mean ± SD (ng/mL)	*p*-Value
Normal	23.32 ± 2.27	NA	22.84 ± 4.36	NA	220.98 ± 2.36	NA
Control	121.61 ± 30.54	^##^ 0.0073	20.72 ± 1.74	0.4177	182.24 ± 9.51	^##^ 0.0025
Kalxetin^®^	169.49 ± 24.59	0.0522	17.14 ± 1.02	* 0.0172	191.54 ± 15.79	0.3600
iPO	99.85 ± 27.35	0.3296	23.80 ± 3.66	0.1976	211.71 ± 17.93	* 0.0374
POF-1	153.98 ± 125.98	0.6485	20.88 ± 2.04	0.9103	192.86 ± 15.92	0.3049
POF-2	196.69 ± 22.52	0.5544	17.68 ± 0.90	* 0.0309	133.55 ± 5.88	* 0.0492
POF-3	81.27 ± 45.02	0.1951	23.71 ± 2.01	0.0662	223.36 ± 19.18	* 0.0155
pPA	123.58 ± 41.20	0.9415	24.43 ± 4.12	0.1718	215.62 ± 28.45	0.0962

NA: Not applicable. ^##^ Very statistically significant as compared with normal group (*p* < 0.01). * Statistically significant as compared with control group (*p* < 0.05). All comparative analyses were based on unpaired *t*-test with Welch’s correction.

## Data Availability

The data presented in this study are available on request from the corresponding author. The data are not publicly available due to the fact that this study is still on going.
